# Social Connections: Transformative Experiences Amid Disruptive Times

**DOI:** 10.1093/geroni/igab046.1609

**Published:** 2021-12-17

**Authors:** Thomas Cudjoe

**Affiliations:** Johns Hopkins University School of Medicine, Baltimore, Maryland, United States

## Abstract

Today many older adults are experiencing intensified social isolation and loneliness as they attempt to “stay safe at home.” The notion, is a stark contrast from our understanding of the importance of social connections on health and well-being. This session highlights: first hand experiences caring for older adults during the COVID-19 pandemic and the implications of social isolation on the health of older adults. The speaker will offer perspectives for ESPO members on the role of community engagement in orienting research agendas, both now (amid the pandemic) and into the future.

